# Using Fluorescent Myosin to Directly Visualize Cooperative Activation of Thin Filaments*^♦^

**DOI:** 10.1074/jbc.M114.609743

**Published:** 2014-11-26

**Authors:** Rama Desai, Michael A. Geeves, Neil M. Kad

**Affiliations:** From the School of Biosciences, University of Kent, Canterbury CT2 7NH, United Kingdom

**Keywords:** Actin, Cooperativity, Myosin, Single Molecule Biophysics, Tropomyosin, Troponin, Muscle Regulation

## Abstract

Contraction of striated muscle is tightly regulated by the release and sequestration of calcium within myocytes. At the molecular level, calcium modulates myosin's access to the thin filament. Once bound, myosin is hypothesized to potentiate the binding of further myosins. Here, we directly image single molecules of myosin binding to and activating thin filaments. Using this approach, the cooperative binding of myosin along thin filaments has been quantified. We have found that two myosin heads are required to laterally activate a regulatory unit of thin filament. The regulatory unit is found to be capable of accommodating 11 additional myosins. Three thin filament activation states possessing differential myosin binding capacities are also visible. To describe this system, we have formulated a simple chemical kinetic model of cooperative activation that holds across a wide range of solution conditions. The stochastic nature of activation is strongly highlighted by data obtained in sub-optimal activation conditions where the generation of activation waves and their catastrophic collapse can be observed. This suggests that the thin filament has the potential to be turned fully on or off in a binary fashion.

## Teaser

**Background:** How calcium regulates thin filament activation is uncertain.

**Results:** Single molecule imaging is used to report on thin filament activation across relevant solution conditions.

**Conclusion:** Two myosin heads are required to activate a thin filament, which occurs as three states, and myosin binding opens up 11 sites for subsequent binders.

**Significance:** This first direct visualization of cooperativity in action exposes how complex systems function.

## Introduction

Striated muscle is a highly organized array of myosin II containing thick filaments inter-digitated with actin containing thin filaments. In the presence of ATP, myosin II interacts with actin to generate force and hence contraction. Calcium switches muscle between contractile and relaxed states through a process mediated by the three protein complex troponin, which locates regularly along the actin filament (1). Calcium binding to troponin triggers a structural reorganization that affects the azimuthal positioning of its partner protein tropomyosin. Structural studies have revealed that this 40-nm coiled-coil protein can occupy three distinct sub-positions along the helical groove of actin (2). Regulation is achieved by tropomyosin interfering with myosin binding to different degrees dependent upon its sub-position. Because tropomyosin forms a head-to-tail continuous filament along actin, a clear structural mechanism for communication of myosin binding along the thin filament is apparent (3). This complex set of interactions permits muscle to be rapidly cycled between active and inactive states in a calcium-dependent manner (1, 4, 5), crucial for the function of vital organs such as the heart.

**FIGURE 1.**
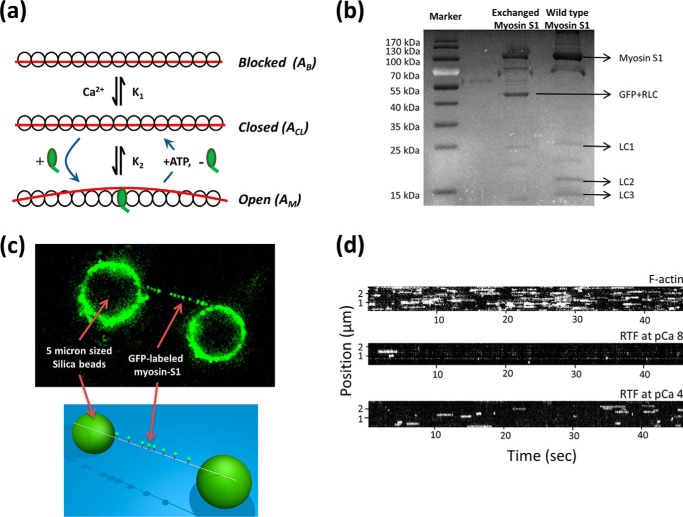
**Detecting activation of thin filament tightropes at the single myosin level.** *a,* three-state model of thin filament activation consists of two transitions as follows: the first between the blocked and closed states is mediated by calcium binding, and only then can myosin bind and transition the thin filament to the open state. ATP binding to myosin in the open state returns the thin filament to the closed state. Myosin potentiates its own binding by activating a regulatory unit on actin that exposes more actin for binding, thus leading to cooperativity. *b,* SDS-polyacrylamide gel comparing exchanged myosin S1 (*middle lane*) with unexchanged myosin S1 (*right lane*). Both myosin S1 preparations were re-purified following papain digestion using gel filtration. *c,* single thin filaments are suspended as tightropes between poly-l-lysine-coated 5-μm beads using microfluidic flow. This permits eGFP-tagged myosin to act as an effector and measure of thin filament activation. The eGFP-labeled myosin can be observed binding to the tightropes using oblique angle fluorescence microscopy. Individual molecules of myosin dissociate once they bind ATP; to represent the attached lifetimes of myosins, the data are represented as kymographs in *d*. In this representation, a slice along the thin filament is projected through time; therefore, line lengths correspond directly to the duration of attachment, and the *y* axis position corresponds to location on the thin filament. No spatial preference of myosin binding was noted in any acquisition indicating there are no “hot spots” of binding. The intensity of the line provides information on the number of bound myosins. For each kymograph in *d*, 1 nm myosin was studied in the presence of 0.1 μm ATP. For all kymographs, the pixel intensity histogram was adjusted for presentation purposes, but during analysis raw data were used.

Calcium alone does not fully activate the thin filament; myosin is also required for full activation (6). A three-state model has been formulated to describe the mechanism of activation, where tropomyosin's three positions correspond to three acto-myosin affinities. The high myosin affinity state (open state) occurs in the presence of calcium and strongly bound myosin, the mid-level (closed state) in high calcium, and the low level (blocked state) in the absence of this cation (Fig. 1*a*). However, these models are still being challenged (7) and refined (8) to attempt to scale *in vitro* observations to those observed in isolated muscle fibers. With no co-crystal structures of myosin bound to the thin filament, precisely how actin, troponin, and tropomyosin interact with myosin is not clear. These details are required to develop a translation to an *in vivo* understanding from the molecular level.

The use of single molecule techniques to understand muscle molecular motors has provided new insights into how these proteins generate motion (9, 10) and force (11). Such studies provide an alternative to bulk ensemble studies that do not provide spatial or mechanical information. Studies of muscle regulation using laser tweezers have revealed that in the absence of calcium near-rigor myosin can activate the thin filament (12). However, a dynamic study at this level across a range of calcium conditions is necessary to provide the detail required for accurate modeling of the complex mechanism of activation. Such a study will be able to define the precise inter-relationship between the activators calcium and myosin and resolve whether myosin binding in one location modulates the binding of distal myosins along the thin filament.

Here, we have developed an *in vitro* model of thin filament activation based on the use of single molecule imaging. We have fluorescently tagged single-headed myosin II to act as both an activator and a reporter of activation. To measure interactions between myosin S1 and actin, we use single reconstituted thin filaments suspended between surface-immobilized pedestals, creating thin filament “tightropes.” These tightropes permit three-dimensional access to the thin filament and eliminate the very likely possibility of erroneous activation from myosins adsorbed to the surface. We are able to directly observe that myosins bind in clusters along the thin filament. These clusters vary in size according to solution conditions thus providing direct evidence of the three states of activation. Calcium only partially activates the thin filament, but in the presence of myosin an activation patch permitting ∼11 myosins to bind locally is formed. These regions of activation can grow, split, diffuse, and catastrophically collapse providing a clear view of how the thin filament activates and also deactivates. All of these observations have been put together into a simple steady-state model of activation, providing a crucial translation from the stochastic single molecule picture of activation to that of ensemble studies.

## EXPERIMENTAL PROCEDURES

### 

#### 

##### Standard Buffer Conditions

The standard assay buffer used during imaging experiments was actin buffer (25 mm KCl, 25 mm imidazole, 1 mm EGTA, 4 mm MgCl_2_, 10 mm DTT, pH 7.4). For all experiments using tropomyosin and troponin (*in vitro* motility and tightrope assays), 100 nm excess troponin and tropomyosin were included in the assay buffer.

##### Protein Preparation

Actin and myosin S1 were prepared from chicken pectoralis skeletal muscle using previously described methods (13). Myosin S1 was prepared using papain digestion of full-length myosin to catalytically cleave off the head domain while retaining its light chains. Digestion was performed by incubating full-length myosin with 1.0 mg/ml papain, 5 mm cysteine, 2 mm EDTA, pH 6.0, at room temperature for 10 min. The reaction was terminated by adding 0.1 m iodoacetic acid. Myosin S1 was subsequently separated from filamentous myosin and S2 by centrifugation. Tropomyosin was expressed in *Escherichia coli* and purified as described previously (14). The three components of troponin were separately expressed in *E. coli* and then combined as cell pellets before dissolving in 6 m urea and sonicating to lyse the cells. Four stepwise reductions in [urea] were performed using dialysis, and the final troponin complex was centrifuged prior to isolation by a two-step ammonium sulfate precipitation, first at 30% to remove contaminants and then 50% to isolate troponin complex. The troponin complex pellet was resuspended in 200 mm NaCl, 10 mm imidazole, 1 mm DTT, 100 μm CaCl_2_ at pH 7 before gel filtration. Fractions of pure troponin complex were then spin-concentrated prior to storage.

##### Labeling Myosin S1 with Fluorescent Regulatory Light Chain

To fluorescently label myosin, we first C-terminally fused smooth muscle RLC^3^ with enhanced GFP (eGFP) genetically. Chicken gizzard smooth muscle RLC was directly synthesized (Epoch Biolabs) and subcloned 5′ to eGFP in a customized *E. coli* expression vector (gift from J. Mason). Maximal soluble protein expression was achieved by cold induction (28 °C) with 1 mm isopropyl 1-thio-β-d-galactopyranoside for 1 h after growth to *A*_600_ 0.6 at 37 °C. Following this, eGFP-RLC was purified by anion exchange chromatography. This construct was then exchanged for the wild type RLC on the myosin S1. To achieve this, 2 μm myosin S1 was incubated with a 2-fold molar excess of eGFP-RLC in exchange buffer (50 mm potassium phosphate buffer, 600 mm KCl, 10 mm EDTA, 2.5 mm EGTA, pH 7.0) and 2 mm ATP at 30 °C for 30 min. The reaction was terminated by the addition of 15 mm MgCl_2_. Exchanged myosin S1 was then dialyzed into myosin buffer (300 mm KCl, 25 mm imidazole, 1 mm EGTA, 4 mm MgCl_2_, pH 7.4) before gel filtration (Sephadex G-200) to separate eGFP-labeled myosin S1 from its unlabeled counterpart. All elutions were analyzed using SDS-PAGE to determine which fractions contained the exchanged myosin S1.

##### In Vitro Motility

Movies of FITC-phalloidin-labeled actin were recorded using the custom-built OAF microscope described below. To calculate filament speeds, the leading edge position of individual actin filaments (*n* >100) was tracked using the MTrackJ plugin for ImageJ throughout each movie. For each condition from *N* flow cells we determined the mean velocity ± S.E. Because we determined velocities for the motile periods of the filament motion, this led to higher speeds at low calcium concentrations than reported elsewhere (12, 15, 16). Three flow cells were used for each condition, and in each a minimum of 30 filaments was examined. Filaments were classified as motile if their velocity was >0.33 μm/s. Measurements were made at 30 °C in motility buffer (51 mm KCl, 25 mm imidazole, 1 mm EGTA, 4 mm MgCl_2_, 10 mm DTT, 0.5% methyl cellulose, pH 7.4) supplemented with oxygen scavengers (12), 100 nm troponin, 100 nm tropomyosin, and when appropriate calcium.

##### Thin Filament Tightrope Assay

Tightropes were created by suspending single thin filaments between silica beads. To adhere the thin filaments to the beads, we first incubated 5-μm sized silica beads (10% w/v) with 340 μg/ml poly-l-lysine solution overnight. 10 μl of this solution was repeatedly washed in ultrapure water (18.2 megohms·cm) before making up to 100 μl in actin buffer. These beads were sonicated before infusion into a custom-built fluidic chamber consisting of a drilled glass slide attached to an amino-silanized coverslip (17). The coverslip was pre-cleaned prior to amino-silanization by ultrasonicating in 100% absolute ethanol followed by 1 m KOH. Beads readily attached to the surface, and a good bead density was determined qualitatively using phase contrast imaging (Nikon Diaphot) prior to attachment of the flow chamber to a programmable syringe pump (World Precision Instruments). Thin filaments or bare actin was added at a concentration of 500 nm by withdrawing the solution from an open microcentrifuge tube. This actin-containing solution was then passed across the surface beads multiple times at a flow rate of 300 μl/min. The resulting tightropes were visualized by using OAF microscopy in motility buffer minus methyl cellulose at 20 °C, with oxygen scavengers, and supplemented with DTT to a final concentration of 100 mm.

The OAF system used here was custom-built into an Olympus IX50 frame. Illumination was achieved using a 17.5× beam expanded 1–20 milliwatts with a 488 nm laser (JDSU^TM^, with custom-written power control software) brought into focus at the back focal plane of an Olympus 100× 1.45 NA objective lens (approximately coincident with the back aperture) using a long focal length anti-reflection-coated plano-convex lens (250 mm, Thorlabs). This created a collimated emerging beam that was tilted by translating the incident beam across the back aperture of the objective using a gimbal mounted mirror (Thorlabs). Just before reaching the critical angle for total internal reflection, an obliquely angled beam was produced that could be tuned to reach the depth of the tightropes. Images were collected by the same objective and then routed to a ×2 magnifier (custom-built into a triplesplit by Cairn Research) via a dichroic mirror (Z488rdc, Chroma) before detection using a DU897 EMCCD camera (Andor, Belfast, UK). Data were analyzed using ImageJ and a collection of custom-written Matlab routines. Two sets of acquisition conditions were used in this study (see Table 1) as follows: 1000 frames at 10 fps and 500 frames at 3.8 fps.

##### Derivation of the Analytical Model

The aim of this model was to recreate intensity histograms (Fig. 4, *b*, *d,* and *f*). We have interpreted these histograms as the sum of the different cluster sizes in a kymograph.

Therefore, we need to calculate the probability of *n* number of myosins being bound at any time. To do this, we used the duty cycle, which is the relative proportion of time a myosin molecule spends attached to the thin filament as shown in Equations 1–3,

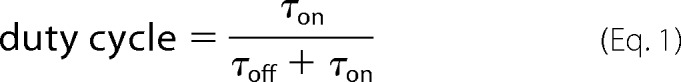


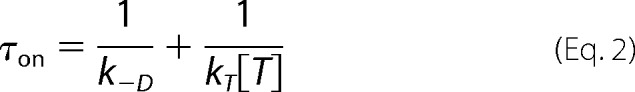


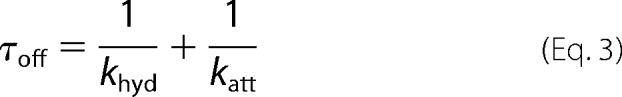

*k*_−*D*_ is the ADP dissociation rate constant when bound to actin; *k_T_* is the second order ATP-binding rate constant; *k*_hyd_ is the ATP hydrolysis rate constant; and *k*_att_ is the attachment rate for myosin to actin. However, *k*_att_ is not easily described when the actin availability is modulated by calcium and myosin binding. To redefine τ_off_ in this context, we have created this simple model based on the classic three-state model (18) combined with the continuous-free chain model (8). This model is shown in Fig. 1*a* and can be described with the following chemical kinetic Scheme 1,

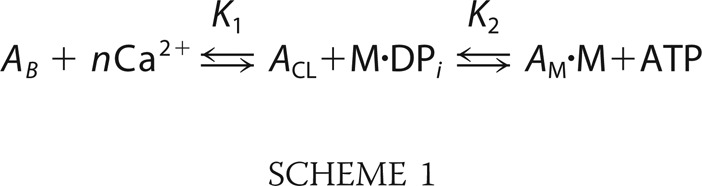

*A_B_* does not bind myosin; *A*_CL_ binds 1–2 myosins; and *A*_M_ will bind “*d*” times more myosin than *A*_CL_. M is myosin, and M.DPi is myosin with hydrolysis products in the active site (denoted M in the equations).

From this the available actin can be derived Equations 4 and 5

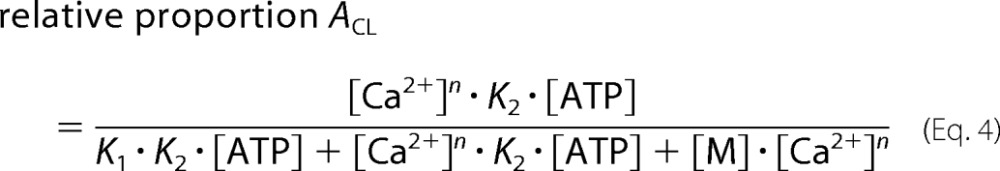


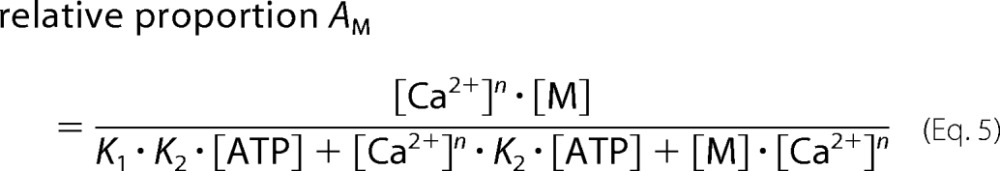
 The first two myosins bind to *A*_CL_; therefore, we must calculate their binding independently as shown in Equation 6,

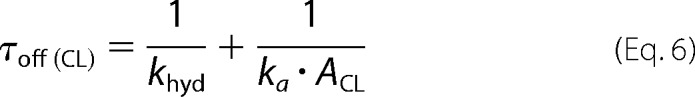
 Subsequent myosins will bind to *A*_M_. However, because a regulatory unit is activated in *A*_M_, *d* times more actin is exposed. This increase in the available actin concentration provides cooperativity as shown in Equation 7,

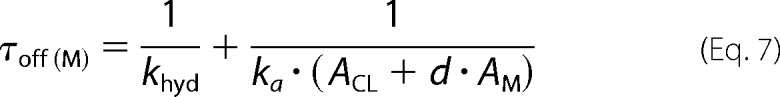
 where *k_a_* is the intrinsic myosin-binding rate.

Each Gaussian component peak in an intensity histogram is equivalent to exactly *n* bound myosins. Therefore, an intensity histogram is the sum of the exact probabilities for each myosin population up to the maximum number of fitted myosins shown in Equation 8,

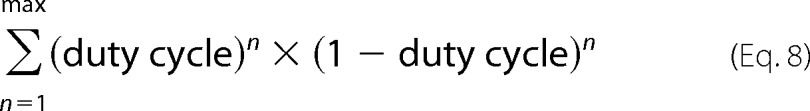
 Where the maximum is determined by fit quality.

Fig. 6*c* shows the data obtained across a spectrum of conditions fitted simultaneously (globally) to this model. The quality of the fits suggest the model and the method are valid. Table 1 shows the parameters from the fits to this model.

**TABLE 1:** **Model parameters determined from global fitting** The fitted parameters used for global fitting are as follows: *k_a_* = the intrinsic myosin binding rate; *K*_1_ = Ca^2+^-induced equilibrium constant for the blocked to closed state transition; *K*_2_ = myosin-induced equilibrium constant closed to open state transition; *k_−D_* = ADP release rate (this value was fixed in the fit); *k_T_* = rate constant for ATP binding; *k*_hyd_ = ATP hydrolysis rate (this value was fixed in the fit); *n*_H_ = Hill coefficient for calcium binding; and *d* = size of the regulatory unit. Dataset i refers to the 10 fps data shown in Fig. 6*c, blue diamonds*; dataset ii is the slower frame rate (3.8 fps) in Fig. 6*c, red squares*. All data were fit at *p*Ca 6.4, and this is the average of that determined from Fig. 2, *a* and *b*.

Parameters used for global	Outcome of the global fitting for data set (i)	Outcome of the global fitting for data set (ii)
*k_a_*	38 s^−1^	47 s^−1^
*K*_1_	0.4 μm	0.4 μm
*K*_2_	85 μm	37 μm
*k_−D_*	500 s^−1^	500 s^−1^
*k_T_*	5 μm·s^−1^	1.4 μm·s^−1^
*k*_hyd_	100 s^−1^	100 s^−1^
*n*_H_	1.22	1.08
ATP	Varied according to dataset	Varied according to dataset
*d*	11.00	11.58

## RESULTS

### 

#### 

##### Fluorescent Tagging of Skeletal Myosin II and Preparation of Thin Filament Tightropes

Skeletal muscle myosin II is double-headed and possesses four light chains. To simplify data analysis, we proteolytically cleaved myosin into its single-headed S1 fragment. For fluorescence imaging, we exchanged one of the two remaining light chains, the RLC, with a bacterially expressed enhanced green fluorescent protein-RLC fusion protein (eGFP-RLC). To select for labeled protein, the exchanged myosin S1-eGFP-RLC (henceforth referred to as myosin) was purified using gel filtration chromatography (Fig. 1*b*). However, it is possible that mis-folding or photobleaching (19, 20) could reduce the effective yield of fluorescent myosin.

##### Observing Thin Filament Regulation

**FIGURE 2.**
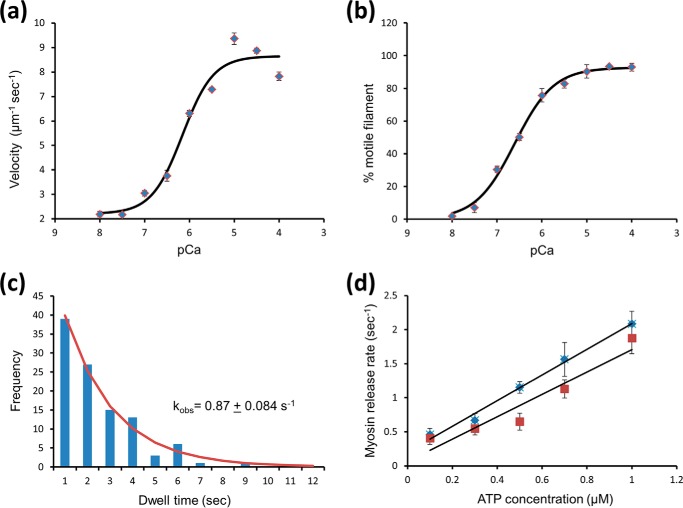
**Confirmation of thin filament regulation.** *a, in vitro* motility sliding velocities of the regulated thin filament were determined using *in vitro* motility, where the thin filament gliding speed over a surface coated with myosin (50 μg/ml) was measured at different calcium concentrations. These data were fitted to a Hill cooperative binding equation providing a Hill coefficient of 1.26 ± 0.46, *p*Ca_50_ of 6.15 ± 0.14, offset velocity 2.2 ± 0.5 μm s^−1^, and maximal sliding velocity 8.65 ± 0.88 μm s^−1^. *b,* percentage of motile filaments *versus p*Ca show a similar increase with calcium with a *p*Ca_50_ of 6.6 ± 0.04. A motile filament was defined (12) as having a mean velocity >0.33 μm s^−1^. *c,* lifetime of attachments from multiple molecules of myosin at 0.8 nm and 0.5 μm ATP with naked actin can be plotted as a histogram. Each lifetime histogram is fitted to an exponential decay providing the detachment rate constant for the solution condition. *d,* plot of the detachment rate constant *versus* ATP concentration shows a linear relationship. The slope of this plot provides the second order ATP-binding rate constant for naked actin (*diamonds*) of 1.88 μm^−1^ s^−1^ and for thin filaments at *p*Ca 4 (*squares*) of 1.64 μm^−1^s^−1^. These values agree well with ensemble studies (39–41).

Individual fluorescent myosins were imaged interacting with single thin filaments reconstituted from chicken skeletal actin decorated with human recombinant cardiac troponin and tropomyosin. These thin filaments were suspended as “tightropes” above a coverslip surface between 5-μm beads (Fig. 1*c*). A high signal-to-noise ratio was achieved using an obliquely angled fluorescent light sheet (OAF) (17), also termed HiLo or variable angle TIRF (21, 22), which illuminated the region in the proximity of the tightropes (Fig. 1*c*). As a first step, we examined the interaction of myosin with bare actin at 0.1 μm ATP. Movies of these interactions were taken, and slices along thin filaments were projected through time to generate kymographs; these representations allow for the frequency and duration of the interaction to be examined. Even with low myosin concentrations (1 nm), binding was extremely frequent (Fig. 1*d, top*). By comparison, a thin filament tightrope at the same concentration of myosin and working at low [Ca^2+^] (*p*Ca 8 = 10 nm Ca^2+^; *p*Ca = −log[Ca^2+^]) showed sparse binding events (Fig. 1*d, middle*). This observation is consistent with the access to myosin-binding sites on the thin filament becoming impeded by the regulatory proteins in a “blocked” state. Corroboratively, *in vitro* motility studies also showed almost complete abolition of thin filament movement at *p*Ca 8 (Fig. 2, *a* and *b*) (12, 15, 23). Such tests were performed in parallel as a control for every preparation of thin filaments used in imaging experiments to ensure the thin filaments were correctly reconstituted. In addition, the second order ATP-binding rate constant was confirmed by studying the ATP dependence of myosin detachment rates at low concentrations (Fig. 2, *c* and *d*). In contrast, at *p*Ca 4 when completely calcium-activated (Fig. 1*d, bottom*), the thin filaments showed much greater myosin binding; however, it is important to note that these filaments did not show as much myosin binding as with bare actin. This therefore indicates that calcium cannot fully activate the thin filament and suggests we are directly visualizing binding to the partially active or “closed” state. Closer inspection of the data in Fig. 1*d* (*bottom*) revealed the fluorescent spots were considerably brighter than at *p*Ca 8, indicative of clustered myosin binding. This clustering suggests that the fully active or “open” state is locally generated by myosin binding to the thin filament in the closed state. To understand this clear interplay between myosin and calcium as activators of the thin filament, we systematically modulated the concentration of myosin, calcium, and ATP. Cluster formation was promoted by increased myosin, increased calcium, and reduced ATP concentrations. To understand the nature of this activation, it was necessary to determine how many myosins were bound per cluster.

##### Quantifying the Number of Myosins in a Cluster

**FIGURE 3.**
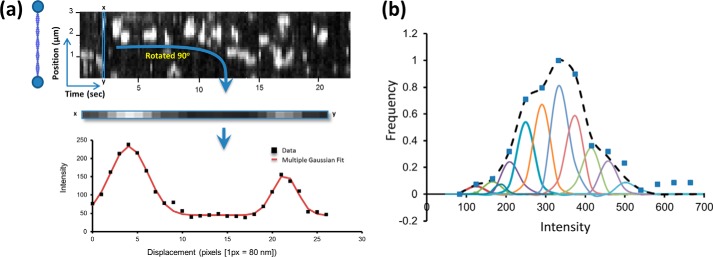
**Analyzing the interactions between myosin and actin.** *a, vertical box* is drawn one pixel wide and scanned along the time axis. Each box contains all of the fluorescence intensity information for a single time point in the movie along the length of a thin filament. In this example, one vertical slice is rotated 90° and plotted as a graph of intensity *versus* position along the thin filament (*lower graph* in *a*). This *graph* shows two peaks with a full width half-maximum of ∼300 nm (>100 actin monomers) as would be expected for a point source. The intensity of the peaks provides information on the number of myosins bound. Therefore, each vertical slice is fitted to a sum of Gaussian distributions with unconstrained intensities (shown as the fit line). The non-zero baseline represents the background noise, which is removed as a consequence of the fitting, which was performed using a custom-written Matlab routine. *b,* peak intensity values from all of the fitted Gaussian distributions were then plotted as a histogram (*blue squares*), and in this case the conditions are 15 nm myosin at *p*Ca 5 with 0.5 μm ATP. As a result of this treatment, the histogram now represents steady-state intensities and has no spatial and temporal information from the kymograph. At low myosin concentrations without regulatory proteins, only single myosins were seen to bind actin resulting in a single peak for the intensity histogram (data not shown). This peak corresponds to the profile of a single eGFP. The next stage of analysis was to determine the number of intensity subpopulations that constitute a multiple myosin intensity histogram. This was achieved by fitting the histogram to multiple Gaussians, each with the standard deviation of a single eGFP until the fit could no longer be improved.

**FIGURE 4.**
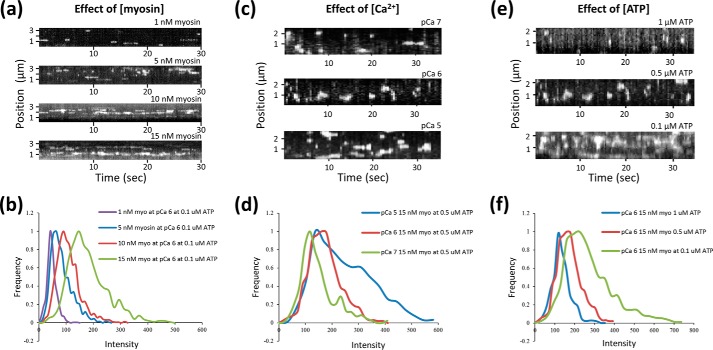
**Thin filament activation responds to myosin, calcium, and ATP concentration.** *a, c,* and *e,* kymographs of myosin interactions with thin filaments at a frame rate of 10 Hz (*a*) and 3.8 Hz (*c* and *e*). As the concentrations of myosin (*a*) and calcium (*c*) are increased, myosin association is favored. This is also seen when the ATP concentration is reduced (*e*). The intensities for each spot per frame were determined using the methodology laid out in Fig. 3*a*, and plotted as histograms (*b, d,* and *f*). The peaks correspond to the most common number of myosins in a cluster, and as more myosins bind the histograms become skewed to higher intensities For accurate data analysis, it was necessary to ensure that clusters were discrete and not overlapping, see Fig. 6*a* for an example of overlapping clusters. In no cases were hot spots of activation observed, where myosins would clearly bind to the same position on the thin filament.

**FIGURE 5.**
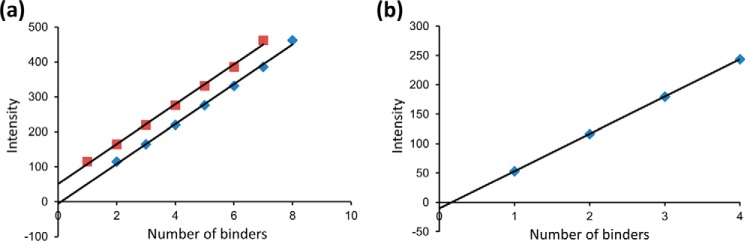
**Two myosin heads are required to activate a regulatory unit.** *a,* intensities of each subpopulation from the fits to the intensity histograms as exemplified in Fig. 3*b* are plotted *versus* the number of myosins within each subpopulation. For the data in *squares,* the first subpopulation is assigned to a single myosin, and subsequent subpopulations are assumed to correspond to single increments in the number of bound myosins. As expected, this results in a linear plot with a slope corresponding to the intensity change per additional eGFP-labeled myosin (56.9 units); however, the *y* axis intercept is non-zero (51.5 units). The data in *diamonds* show how the linear fit is made to pass through zero by simply assuming the first subpopulation corresponds to two myosin heads. This indicates that minimally two myosins are required to activate the thin filament. *b,* in nonactivating conditions (15 nm myosin, *p*Ca 7, 0.5 μm ATP), the intensity values for each myosin binding subpopulation were plotted against a linear scale of binders starting with a first population of a single myosin. This linear regression fit to the data indicates an intensity change per eGFP-labeled myosin of 63.5 intensity units. Unlike the data shown in *a,* re-registration was not required to bring the ordinate intercept close to zero. This indicates that first intensity subpopulation corresponds to a single myosin population in nonactivating conditions.

Given that our observations all contained a time dimension, we chose to analyze the data from a steady-state perspective. This also provided a means to compare single molecule observations with ensemble studies. To calculate the number of myosins bound for all clusters on individual thin filaments, we analyzed kymographic data directly by taking consecutive vertical slices through the kymograph and fitting each one with a custom-written (Matlab) multiple Gaussian fitting routine (Fig. 3*a*). The intensities from these fits were then re-plotted as histograms to represent the distribution of myosin cluster intensities found in the steady state (Fig. 4, *b*, *d,* and *f,* and illustratively in Fig. 3*b*). The bin sizes for these histograms were independently determined using kernel density estimation (24). These histograms were deconvolved by fitting to a sum of Gaussian distributions fixed only by the standard deviation that was determined by the fit to a single eGFP (25). Because each of these Gaussian distributions corresponds to another bound myosin per cluster, plotting the mean position as a linear series provided an expected straight line (Fig. 5*a*). The slope of this line provides the intensity change per myosin bound, equivalent to the intensity of a single eGFP-labeled myosin. Because the background is removed during the kymograph fitting phase (Fig. 3*a*), this graph would be expected to extrapolate through zero; however, this plot has a non-zero offset. To reconcile this, the assignment of the first peak was shifted to two myosins causing the graph to now extrapolate through zero. We found that for all conditions studied where the thin filament was activated (determined by myosin clustering), it was necessary to shift the assignment of the first intensity peak to two myosins. At *p*Ca 7 this was not the case, and the plots passed close to zero with one myosin as the first peak (Fig. 5*b*). This suggests that to activate the thin filament, the binding of two myosin heads is required, and once activated many more myosins accumulate in the active region.

##### Submaximal Thin Filament Activation

**FIGURE 6.**
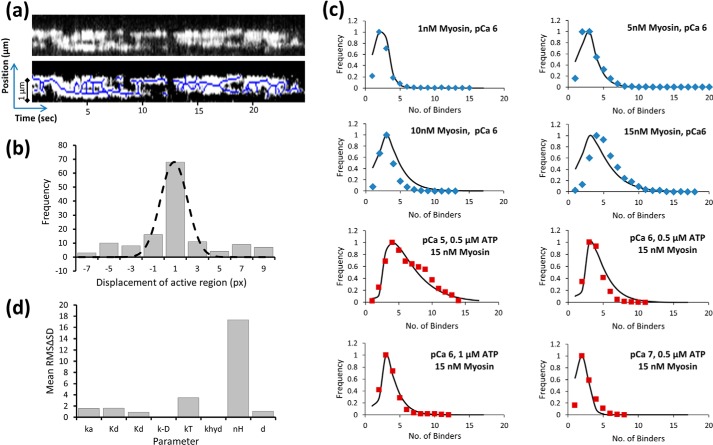
**Complexity of thin filament activation.** *a,* kymograph in sub-maximal activation conditions (*p*Ca 6, 15 nm myosin, 0.1 μm ATP) shows a continuous patch of activation that moves both toward the plus and minus ends of actin. The *lower kymograph* has the peak intensities labeled (using ImageJ skeletonize); this allow a clearer view of how these active regions collide and collapse catastrophically. The skeletonization was used only for visual purposes and not for the fitting used in *b*. This image is the first real time single molecule view of how the thin filament both activates and deactivates. *b,* center of a number of active regions was analyzed by measuring their positional displacement over a single frame and plotted as a histogram. This histogram follows a Gaussian distribution indicative of diffusion; the mean position (−0.88 ± 1.11 (S.D.) px) suggests the diffusion is unbiased. One pixel = 80 nm. *c,* global fitting of the intensity histogram data (Fig. 4, *b*, *d,* and *f*) across a number of conditions. The data in *blue diamonds* (10 fps) was obtained at a faster frame rate than those in *red squares* (3.8 fps) and were therefore fit separately; the results in either condition provide the same value for *d* of 11. The quality of the fits both validate the choice of model and provide an activation distance for the open state of 11 myosin-binding sites on actin. The remaining parameters used in this model are provided in Table 1. *d,* sensitivity was tested for each the parameters stated in Table 1 used in the fitting for *c*. The value for each parameter was first halved, and the resulting ΔS.D. of the fit was squared and then summed with the ΔS.D. obtained when the same parameter was doubled. This root of this value is plotted as the root mean square ΔS.D. value (or sensitivity) for each parameter. A more sensitive value indicates the parameter is fit with better precision.

With a better understanding of the relationship between the thin filament activators, we sought to explore the processes prior to full activation and also relaxation by studying thin filaments in sub-maximal activation conditions as follows: low ATP, a Ca^2+^ concentration just above the *p*Ca_50_ (∼*p*Ca 6.2; Fig. 2) and a high myosin concentration (15 nm). In these conditions, we were able to see the formation of clusters that contain much greater numbers of myosins (up to 12 myosins per cluster); surprisingly, however, these clusters were seen to move along the thin filament as myosins would bind and leave (Fig. 6*a*). To quantify, if there was any directional bias to the motion of these active regions, we determined their median position and plotted its displacement between each frame. Plotting a histogram of the displacements from three separate thin filaments (Fig. 6*b*) gave a Gaussian distribution that was centered on 0.9 pixels (px) but with a standard deviation of 1 px. Therefore, this distribution is indistinguishable from an unbiased random walk and indicates that activation is not directional. However, because we could not establish the polarity of the actin filaments, it is possible that by summing the displacements between filaments any bias to the walk direction would cancel out. We therefore studied the displacements from each filament individually and found no directional bias (data not shown). Therefore, myosin can bind and release from either end of the activated region with equal probability. Also seen in Fig. 6*a* are a number of other features. It can be seen that activated regions appear to merge and split. This behavior may contain information about the longer distance effects of activation. We are presently engaged in quantifying this complex behavior. Fig. 6*a* also shows collapse events, consistent with our expectation that at least two myosins are required to sustain activation.

##### Quantitative Modeling

To provide a quantitative description of the activation process, we formulated the following descriptive model similar to the three-state model (18): without calcium, the thin filament is in an inhibited state (blocked state, *A*_B_), and we observed no myosin binding. With calcium, the thin filament partially activates (closed state, *A*_CL_), permitting the binding of myosins. If two myosins bind in close enough proximity, then a region of the thin filament is fully activated (open state, *A*_M_) resulting in the potentiated binding of myosin. Because myosin binding is not uniform across the thin filament at high calcium concentrations, calcium alone does not allow for population of the open state. A similar picture has been suggested both by ensemble kinetic (18, 26, 27) and structural studies (2, 28). However, in this study, we were able to directly visualize these states. The three-state activation model we present here (see Scheme 1 and Fig. 1*a*) is not limited to the unit length of tropomyosin (8). Based on our observations, myosin cannot bind to *A*_B_; only 1–2 myosins bind to *A*_CL_, but multiple myosins bind to *A*_M_ as a consequence of myosin unblocking tropomyosin's inhibition. Although the majority of myosin will have ADP and inorganic phosphate in the active site, due to the low ATP concentrations used there will be present a small population of rigor heads.

The relative proportions of *A*_B_, *A*_CL_, and *A*_M_ were used to determine the free actin available for myosin binding. *A*_M_ binds *d* more myosins than *A*_CL_, where *d* is the amount of actin exposed as a consequence of myosin binding, *i.e.* the regulatory unit, and therefore *A*_M_ represents the concentration of active regulatory units. Whether *d* represents contiguous actin monomers is uncertain because it is not clear how closely neighboring myosins pack onto the actin filament. However, our definition of the regulatory unit purely reflects the increase in available actin following the binding of the first two heads, and therefore it does not invoke a physical size component. To generate myosin binding probability, distribution plots equivalent to the intensity histograms in Fig. 4, *b*, *d,* and *f*, the probability of myosin binding is calculated using the attached lifetime ratio or duty cycle. The probability of exactly *n* myosins bound to the thin filament is given by (duty cycle)*^n^* × (1 − duty cycle)*^n^*. The sum of the probabilities for increasing numbers of myosin molecules bound provides the myosin binding probability distribution plot. This expression was used to fit the intensity histograms globally across a range of conditions (Fig. 6*c* and Table 1). The quality of the fits is excellent, thus indicating this simple model is highly effective at predicting the nature of thin filament activation. Parameter sensitivity is shown in Fig. 6*d*. The Hill coefficient and second order ATP-binding constant show the greatest levels of sensitivity suggesting these are the best fit parameters. As expected, the least well fit parameters are insensitive to the conditions of this study, the rate constant for ADP release from acto-myosin and myosin's ATP hydrolysis rate constant. Therefore, activation is a two-step process with calcium partially activating the thin filament and then myosin opening a region on the thin filament that permits ∼11 additional myosins to bind.

## DISCUSSION

The mechanism by which muscle contraction is controlled provides a paradigm for understanding the processes of cooperativity and biological systems more generally. By using direct imaging of single myosin S1 (simply termed myosin here) molecules interacting with suspended thin filaments, we are able to determine when and where the thin filaments are active. We show that even when the calcium concentration is 2 orders of magnitude above the *K_d_* values the thin filament only partially activates; substantially fewer myosins are bound than with bare actin (actin with no regulatory proteins present). It is clear, however, that myosin binding occurs in clusters in such partially active conditions. This confirms that myosin propagates its own binding by creating locally fully active regions known as regulatory units. Quantification of the number of myosins bound in a cluster across a range of myosin, calcium, and ATP conditions have been modeled using a three-state binding mechanism. From this analysis, we are able to suggest that at least two myosin heads are required to activate a regulatory unit of the thin filament that can accommodate ∼11 myosins. We are also able to show that in sub-maximal activation conditions, the regulatory units fuse to form larger active regions capable of moving along the thin filament, suggesting that *in vivo* the thin filament may be entirely switched on or off.

### 

#### 

##### Calcium, Myosin, and Actin as Modulators of Activation

**FIGURE 7.**
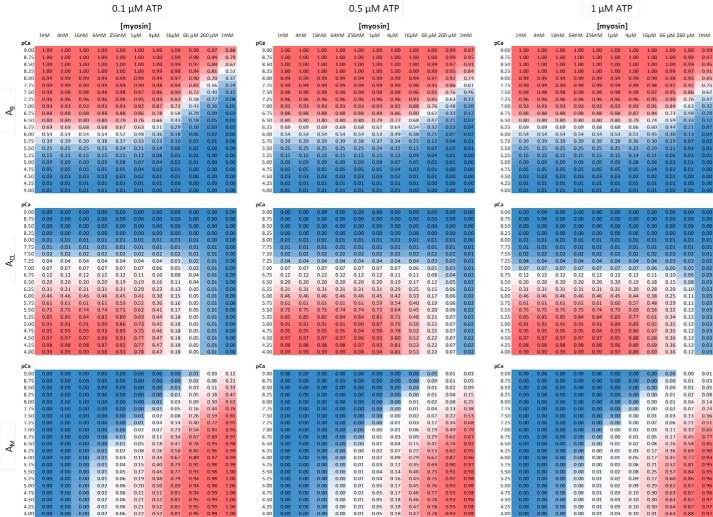
**Relative populations of the blocked (*A*_B_), closed (*A*_CL_), and open (*A*_M_) states across solution conditions.** Using the parameters defined in Table 1 from the fits in Fig. 6*c,* it is possible to study the effects of solution parameters on the populations of the three states in the model (Fig. 1*a*). Each of the nine panels here is color-coded to represent the highest population in *red* and lowest in *blue* (actual values are in each box). The *x* axis for each panel represents [myosin] ranging from 1 nm to 1 mm, and the *y* axis is *p*Ca, ranging from 9 to 4. The ATP concentration is stated above each column. It can be seen that calcium triggers the transition from blocked to closed, and then myosin is required to further push the thin filament into the open state. This is counter-balanced by the [ATP], which reduces the population of the open state, instead favoring the closed state. The population of the open state is still an underestimate of the true available actin concentration because *A*_M_ represents the concentration of activated regulatory units. This is the basis of the cooperative binding of myosin in this system.

Global fitting of intensity histograms (Fig. 6*c*) has allowed determination of numerous parameters for our interpretation of the three-state activation model (Fig. 1*a*). By fixing these parameters, we are now able to explore the populations of the blocked, closed, and open states across a range of myosin, ATP, and calcium concentrations. Fig. 7 is a multi-panel view of the relative population of these states. It can be seen that as the calcium concentration is increased, the blocked state is depopulated in favor of the closed state. At low [myosin], no population of the open state is possible; however, increasing concentrations of myosin lead to greater formation of the open state. This occurs at the expense of the closed state, such that at very high concentrations of myosin the population of the closed state is nearly eliminated. Such high concentrations of myosin could be present *in vivo* where the myosin is closely packed next to the thin filament. However, these calculations were performed for the experimental conditions used in this study, *i.e.* low [ATP]. As the [ATP] is raised, the closed state reappears. At physiological ATP (>1 mm), the myosin concentration needs to reach ∼20 mm to deplete the closed state (data not shown).

##### Two Heads Are Required to Activate the Thin Filament

The role of the two heads of full-length myosin has remained an enigma for several decades (29–32). Single molecule experiments have indicated that only with both heads present is the full displacement realized (33, 34). However, in the context of activation, we have shown here that a second head may play a role in activating the thin filament. A reduction in force within muscle fibers has been noted for single-headed constructs (29), although it is difficult to extract whether this was due to changes in unitary force, which again highlights the difficulties of understanding such a complex system. This work does not demonstrate that this is the role of the second head, rather that two heads are required to activate the thin filament. The required second head, *in vivo*, may reside on a second double-headed myosin molecule, and therefore, our data minimally indicate that the binding energy gained from two myosin heads is required to activate a regulatory unit on the thin filament. How myosin activates the thin filament is uncertain. Does it function as a steric block to the relaxation of the tropomyosin and therefore only binds during thermal excursions of tropomyosin to the open state? Or, alternatively, once bound to the closed state does myosin alter the tropomyosin-binding energy landscape on actin, thus favoring the transition to the open state? This assay offers the potential to answer such questions.

##### 11 Myosins Are Accommodated in a Regulatory Unit

Tropomyosin's persistence length limits the bend angles accessible through thermal energy, estimated to be equivalent to the contour length of a monomer (35). Therefore, as the thin filament is activated a region beyond the bound myosin(s) will be available for other myosins to bind. In our model this is the sole origin of the cooperativity during activation. Even calcium binding was free-fitted and showed very little cooperativity (Table 1). Because monomeric tropomyosin assembles head-to-tail along the thin filament, it is conceivable that activation extends beyond a tropomyosin monomer. Our data here suggest that ∼11 myosins can bind to the thin filament following its activation by the first two binders; hence, the regulatory unit size is 11. Because the point spread of a single fluorophore extends over ∼50 actin monomers on one strand, equivalent to ∼7 end-to-end tropomyosin monomers, we cannot determine whether these myosins bind on adjacent actin monomers. Also, given that this is a cooperative process, the binding of subsequent myosins would in turn activate more of the thin filament leading to explosive growth of activation. To determine the precise location of myosin binding would require application of stochastic super-resolution methods in combination with our thin filament tightrope assay. However, in a dynamic system where myosin is rapidly binding and releasing, such methods are extremely challenging. Nonetheless, determining the physical size of the regulatory unit is of great value, and particularly interesting could be that the regulatory unit size may change with solution conditions. It is also possible that activated regions include myosins binding to the opposite strand of actin; however, the probability that such random occurrences could lead to the data seen here is unlikely without a coordinated mechanism for cross-talk between strands. A recent structural study has indicated that troponins may be able to interact across actin (36); however, much more evidence will be required for such a conclusion to be drawn. Therefore, because 11 myosins exceed the number of sites occluded by a single tropomyosin, the regulatory unit most likely extends beyond the monomer boundary in agreement with previous studies (42).

The use of low ATP concentrations in this study also raises the issue of rigor activation *versus* active myosin activation. How myosin activates the thin filament is envisaged as steric unblocking, where myosin binding unblocks the accessibility of other myosins to bind. Previous data indicate that activation of the thin filament follows this process and simply depends on the bound lifetime of myosin (12). In this study, we find no difference between the bound lifetimes of myosin on regulated or bare actin (Fig. 2*d*), suggesting that the regulatory proteins are not forcing myosin from the actin. Therefore, there is no evidence to suggest that myosin in rigor or with ADP·P_i_ bound alters the mechanism of thin filament activation. By using very low [ATP], we permit the process to be detected using conventional fluorescence; however, experiments are underway to observe this process more quickly to resolve individual myosins binding to and releasing from the thin filament at high [ATP] and [myosin].

##### Extended Active Regions

Thin filament activation is inherently a stochastic process requiring the local binding of a calcium ion and two myosin heads, which provides a substantial entropic barrier to activation. We see in sub-maximal activation conditions the formation of moving activated regions. This peek into the complex processes of activation shows that until enough myosin and calcium are present, the thin filament is on the verge of turning completely off. We see this in Fig. 6*a*, where regions of activation suddenly collapse. One would expect that with greater myosin and calcium concentrations, these regions will grow and unify to turn on the entire thin filament. Such scenarios have recently been stochastically modeled (37) and are in complete agreement with our data here. Further analysis of activation in sub-maximal conditions is currently ongoing. The close proximity of full-length myosin to actin *in vivo* leads to a very high local concentration, overcoming some of the entropic barrier to activation, and hence the dual requirement for calcium to activate the thin filament. A very important aspect of normal muscle function is its ability to relax. Nowhere is this more evident that in the heart, where cardiac disease has been hypothesized to result from an inability of contraction to completely cease (38); the data presented here provide evidence that this occurs as a catastrophic collapse of activation once the full-length myosin and calcium together cannot sustain the active state. Therefore, using the method developed here, we have been able to visualize cooperativity enabling the fundamental processes that underlie muscle relaxation to be studied. It is anticipated that this approach will directly impact our understanding of diseases such as cardiomyopathies, the molecular pathology of which are currently highly debated.

## Footnotes

3 The abbreviations used are:

RLC: regulatory light chain
eGFP: enhanced GFP
OAF: oblique angle fluorescence
fps: frames/s
px: pixel.
